# Urate Nephropathy from Tumor Lysis Syndrome in an Undiagnosed Case of Prostate Cancer

**DOI:** 10.3390/curroncol28010046

**Published:** 2021-01-13

**Authors:** Sidra Javed

**Affiliations:** Department of Medicine, Division of General Internal Medicine, University of Calgary, Calgary, AB T2N 1N4, Canada; sidra.javed@albertahealthservices.ca

**Keywords:** thrombotic thrombocytopenia, hemolytic uremic syndrome, tumor lysis syndrome, urate nephropathy, hemodialysis

## Abstract

Prostate cancer can masquerade as just normocytic anemia and thrombocytopenia, thrombotic thrombocytopenic purpura (TTP), hemolytic uremic syndrome (HUS), or tumor lysis syndrome (TLS). We are reporting an intriguing case of metastatic prostate cancer which remained undiagnosed until the patient showed signs of tumor lysis syndrome (TLS), leading to urate nephropathy requiring urgent hemodialysis. Tumor lysis syndrome is an oncological emergency but an exceedingly rare complication in non-hematological malignancies, including prostate cancer. It is challenging to recognize features of TLS in a case such as this with an unknown diagnosis. In the case of an established diagnosis of malignancy, however, checking baseline renal function, uric acid, lactate dehydrogenase (LDH), potassium, and phosphate to monitor for TLS as well as considering urate lowering therapy can help prevent adverse outcomes.

## 1. Introduction

An 80-year-old man was admitted to our medical service with constitutional symptoms (40 lbs weight loss over 2–3 months, low appetite, and fatigue). His past medical history was significant for hypertension, gastroesophageal reflux disease, benign prostatic hyperplasia, and partial gastrectomy due to peptic ulcer disease in the 1960s. He had a brief admission to a teaching hospital with idiopathic pericarditis 2.5 months ago. A month later, he had an uncomplicated elective right hip arthroplasty. Approximately three weeks before his current presentation, he was admitted with similar symptoms of weight loss and normocytic anemia, Mean Corpuscular Volume 90 fL (*N* 82–100 fL) with an Hgb of 68 g/L, and a mild pre-renal acute kidney injury which improved with blood transfusion and intravenous fluids. An upper endoscopy was unremarkable, and a colonoscopy was deferred due to an unclear picture of lower gastrointestinal bleed and a normal colonoscopy from 2013. An ultrasound of the right hip demonstrated a 10 × 3 cm collection, which was thought to be a seroma due to lack of infectious symptoms and signs.

He continued to experience similar symptoms and presented to the hospital once again. On admission, the examination was only remarkable for mild splenomegaly on palpation. There was no lymphadenopathy noted.

His hemoglobin was 57 g/L, leukocytes 6.2 10E9/L, and platelets 129 10E9/L. His electrolytes and creatinine were normal (Table 1). Hemolysis workup including, haptoglobin, bilirubin, fibrinogen, and direct antiglobulin test (DAT) was negative. Initial peripheral blood smear was unremarkable; particularly, no schistocytes were seen. His relative reticulocytes were 3% (*N* 0.2–2%). He had some rare tear drop cells, myelocytes, and metamyelocytes along with nucleated red cells with red cell width (RDW) ranging between 17–18% (*N* 11–16%) on subsequent blood smears. A diagnosis of thrombotic thrombocytopenic purpura was ruled out by the input of hematology colleagues. Thyroid stimulating hormone, vitamin B12, folate levels, and lipid panel were in the normal range. Liver enzymes were notable for elevated alkaline phosphatase (ALP) and gamma-glutamyl transferase (GGT) at 371 (30–145) U/L and 89 (11–63) U/L, respectively. Infectious and autoimmune workup, particularly common viral hepatitides and HIV serologies, were unremarkable. C-reactive protein and ferritin levels were elevated (Table 1). Myeloma screen including serum and urine protein electrophoresis did not show monoclonal protein. Kappa to lambda ratio was reported to be normal (1.0). Antinuclear antibodies (ANA) level was weakly positive (1:80). Complement levels (C3 and C4), extractable nucleic acid (ENA), anti neutrophilic cytoplasmic antibodies (ANCA), and anti glomerular basement membrane (anti-GBM) were all within normal limits. He was assessed by our rheumatology service for a possible autoimmune etiology given the recent pericarditis episode. In the absence of any specific immunological marker, an autoimmune disorder was considered less likely.

A prior computed tomography (CT) scan of his chest for pulmonary embolism during his pericarditis episode did not demonstrate pulmonary embolism, lymphadenopathy, or any osseous abnormality. A CT abdomen and pelvis on presentation indicated mild splenomegaly with the splenic index of 780 (*N* < 480), but no masses and intra-abdominal lymphadenopathy were identified.

Due to the profound elevation of inflammatory markers, an ultrasound-guided right hip collection aspiration was performed, which did not show growth of any organism after 4 days.

On day 5 of his admission, he started to develop Acute Kidney Injury with oliguria and hyperkalemia requiring urgent hemodialysis. Urine microscopy revealed numerous urate crystals and granular cast but no dysmorphic red cells. Creatinine kinase (CK) was normal, and urate level turned out to be at 1226 µmol/L. The rest of the tumor lysis syndrome workup is shown in Table 1.

An unenhanced CT of his chest was ordered to look for mediastinal and hilar lymphadenopathy secondary to hematological malignancy as a potential reason for suspected tumor lysis syndrome. To our surprise, poorly defined multiple lucent lesions were observed in thoracic vertebrae suspicious for metastatic disease. While waiting for a bone scan, we then performed a bone marrow biopsy that demonstrated 40% of the marrow space area being involved by metastatic adenocarcinoma consistent with prostatic origin. Immunohistochemistry testing confirmed that the neoplastic cells were positive for Prostate Specific Antigen (PSA) and *NKX3.1*.

## 2. Discussion

Prostate cancer is the most common cancer among Canadian men (excluding non-melanoma skin cancers). It is the third leading cause of death from cancer in men in Canada [1]. It can masquerade as a clinical picture of just anemia and thrombocytopenia due to infiltration of bone marrow [2], thrombotic thrombocytopenic purpura (TTP), complement mediated hemolytic uremic syndrome [3], and/or tumor lysis syndrome [4].

Tumor lysis syndrome (TLS) is an oncological emergency but an exceedingly rare complication in non-hematological malignancies, including prostate cancer [4]. In literature, there are limited cases of TLS secondary to metastatic prostate cancer with and without treatment. Our literature review for “prostate cancer” and “spontaneous tumor lysis syndrome” revealed four cases of treatment naïve prostate cancer associated with TLS [3,5,6,7]. Out of these four cases, three had a pre-existing diagnosis of prostate cancer, and only one was a diagnostic mystery [3] such as ours. TLS is characterized by hyperuricemia, hyperkalemia, hyperphosphatemia, and hypocalcemia. According to Cairo and Bishop, the most widely accepted diagnostic criteria, TLS is defined as the presence of at least two or more of the following metabolic abnormalities (Table 2) within 3 days before and 7 days after initiation of chemotherapy in the face of adequate hydration and use of uric acid lowering agent [8].

Clinical tumor lysis syndrome (CTLS) is defined as the presence of laboratory tumor lysis syndrome (LTLS) and any one or more of the criteria mentioned in Table 3.

Our case met LTLS and CTLS criteria and helped to navigate towards a diagnosis. TLS occurs due to the rapid destruction of tumor cells and purine catabolism in response to cytotoxic therapy or the high turnover of highly proliferative tumors. The most common mechanism of acute kidney injury in tumor lysis syndrome is very well described crystal-induced tubulopathy (blockage of distal tubules by precipitation of crystals). When the renal excretory capacity exceeds, hyperuricemia develops, and an acidic pH promotes uric acid crystal formation that subsequently leads to intraluminal obstruction. Recently uric acid has also been hypothesized as a potential inflammatory mediator that might accentuate AKI in addition to crystal mediated renal injury [9].

The principal feature of the successful management of TLS is to identify high-risk patients and institute prophylactic strategies to reduce the aggressiveness of clinical manifestations. Solid malignancies are considered low risk to develop tumor lysis; however, there are several other factors that indicate severity. Aggressive hydration with urine alkalinization is a key to prevent urate precipitation and subsequent kidney injury. Allopurinol, a xanthine oxidase inhibitor, is a commonly used medication in this setting. Allopurinol reduces the formation of new uric acid that ultimately lowers the risk of obstructive uropathy in patients with malignant disease at risk of TLS [8]. Allopurinol, however, does not prevent tubular injury from uric acid that has been pre-formed before the initiation of this drug [8]. Patients with either the presence of laboratory TLS and/or clinical TLS would be better candidates for rasburicase therapy for prophylaxis and/or therapy of hyperuricemia. Rasburicase is a recombinant form of urate oxidase and converts uric acid to allantoin, which is five to ten times more soluble in urine than uric acid [10,11].

In our patient, in view of a rapid deterioration of metabolic changes and an unclear diagnosis, hemodialysis was considered the best approach. One can argue that hyperphosphatemia and hyperkalemia both could be a complication of renal failure itself, but there was no other clear etiology identified for AKI. Significant hyperuricemia as well as a primary reason for AKI in the absence of unstable hemodynamics or addition of new medications were unexplainable otherwise. Later, a nuclear bone scan revealed diffuse skeleton metastatic deposits including bilateral proximal humeri, bilateral ribs, bilateral proximal femora, thoracic spine, and, to a lesser degree, lumbar spine. This significant burden of the disease supports the possibility of spontaneous tumor lysis syndrome.

The finding of splenomegaly in our patient remained unexplained; however, this could be related to extramedullary hematopoiesis with the leucoerythroblastic picture. With normal iron indices, this is potentially indicative of myelophthistic anemia due to infiltration of bone marrow with metastatic cells and fibrosis [12]. This is only a speculation, as only one out of four blood smears showed rare tear drop cells with immature granulocytes.

Unfortunately, there was no previous imaging available to compare splenic size. Elevated ferritin level along with splenomegaly made us suspect hemophagocytic lymphohistocytosis (HLH), but with the history of repeated blood transfusions and normal lipid panel, it was considered less likely. Additionally, we considered to look for a secondary cause, e.g., infection or malignancy, as a potential reason for HLH if that was a cause for our patient’s clinical picture. On dedicated images for staging, no metastatic disease was identified in solid organs.

Our patient’s prostate specific antigen (PSA) level was found to be elevated at 36.8 µg/L (*N* 0.0–6.5). His follow-up biochemical picture was clouded by hemodialysis required for anuric toxic Acute Tubular Necrosis (ATN), which corrects metabolic abnormalities temporarily. He required only two runs of hemodialysis. Most of his biochemical abnormalities improved over the next 2 weeks after the initiation of androgen deprivation therapy (Table 1). Repeat urinalysis did not report uric acid crystals. PSA level also declined to 5.6 µg/L with the initiation of abiraterone and prednisone within a month of his discharge from the hospital with a diagnosis of castration-naïve metastatic prostatic cancer. His outpatient investigations within the next six months showed normal creatinine level (average values shown in Table 1 in January–June column). Unfortunately, urate level was never repeated in the outpatient setting; however, no urate crystals were identified on urinalysis at each visit.

## 3. Conclusions

In summary, this was an intriguing case with an atypical presentation of a primary malignant process that remained undiagnosed for a month. It is challenging to recognize features of TLS in a case such as this with an unknown diagnosis. Our case emphasizes the fact that TLS, though exceedingly rare, is a consideration in metastatic prostate cancer. In the case of an established diagnosis of malignancy, however, checking baseline renal function, uric acid, lactate dehydrogenase (LDH), potassium, and phosphate to monitor for TLS as well as considering urate lowering therapy can help prevent adverse outcomes.

## Figures and Tables

**Table 1:** Lab values showing a comparison with the values from previous admissions. (-) denotes unavailability of the lab test.

Blood Test	June 2019	August 2019	September 2019	10 October 2019	14 October 2019	30 October 2019	4 November 2019	6 November * 2019	18 November ** 2019	January–June 2020
Hgb _g/L_ (120–140)	148	135	87	68	81	55	72	84	79	92
Platelets _10E9/L_ (150–400)	219	147	103	118	148	128	72	44	72	179
Cr _umol/L_ (50–120)	112	100	101	217	107	131	430	706	95	91
CRP _mg/L_ (0.0–8.0)	-	22.1	-	92	37.1	55.6	-	266.9	-	-
Ferritin _ug/L_ (30–500)	-	-	-	8978	-	14785	-	18258	-	-
K _mmol/L_ (3.5-5.0)	4.6	4.1	4.2	5.4	4.1	4.4	5.3	6.4	3.7	3.8
Ca _mmol/L_ (2.10–2.60)	-	-	-	2.38	-	2.39	2.27	-	-	2.50
PO4 _mmol/L_ (0.80–1.50)	-	-	-	1.32	-	1.37	2.39	-	-	-
Urate _umol/L_ (210–490)	-	-	-	-	-	-	1226	-	-	-
LDH _U/L_ (100–235)	-	235	-	387	-	609	678	484	287	173

* indicates dialysis followed by initiation of androgen deprivation therapy within a week. ** discharged home. Hgb-hemoglobin, Cr-creatinine, CRP- c-reactive protein, K-potassium, Ca-calcium, PO4-phosphorous, LDH- lactate dehydrogenase.

**Table 2:** Cairo and Bishop laboratory criteria for tumor lysis syndrome (TLS) (laboratory tumor lysis syndrome, LTLS).

Biochemical Marker	Values
Uric acid	x ≥ 476 μmol/L or 25% increase from baseline
Potassium	x ≥ 6.0 mmol/L or 25% increase from baseline
Phosphorous	x ≥ 2.1 mmol/L (children), ≥1.45 mmol/L (adults) or 25% increase from baseline
Calcium	x ≤ 1.75 mmol/L or 25% decrease from baseline

**Table 3:** Cairo and Bishop clinical criteria for TLS (clinical tumor lysis syndrome, CTLS).

**Cairo and Bishop Clinical Criteria for TLS (CTLS).**
(1) Creatinine ≥ 1.5 × ULN (age > 12 years or age adjusted)
(2) Cardiac arrhythmia/sudden death *
(3) Seizure *

* Not directly or probably attributable to a therapeutic agent, ULN—upper limit of normal.

## Data Availability

No new data were created or analyzed in this study. Data sharing is not applicable to this article.
